# Retraction Note: TLE3 represses colorectal cancer proliferation by inhibiting MAPK and AKT signaling pathways

**DOI:** 10.1186/s13046-021-02079-2

**Published:** 2021-09-01

**Authors:** Run-Wei Yang, Ying-Yue Zeng, Wen-Ting Wei, Yan-Mei Cui, Hui-Ying Sun, Yue-Long Cai, Xin-Xin Nian, Yun-Teng Hu, Yu-Ping Quan, Sheng-Lu Jiang, Meng Wang, Ya-Li Zhao, Jun-Feng Qiu, Ming-Xuan Li, Jia-Huan Zhang, Mei-Rong He, Li Liang, Yan-Qing Ding, Wen-Ting Liao

**Affiliations:** 1 Department of Pathology, Nanfang Hospital, Southern Medical University, Guangzhou, 510515 Guangdong China; 2 Department of Pathology, School of Basic Medical Sciences, Southern Medical University, Guangzhou, Guangdong China; 3 Guangdong Provincial Key Laboratory of Molecular Tumor Pathology, Guangzhou, Guangdong China; 4 Guangdong Provincial Key Laboratory of Gastroenterology, Department of Gastroenterology, Nanfang Hospital, Southern Medical University, Guangzhou, Guangdong China; 5 Department of Pathology, Nanfang Hospital and School of Basic Medical Sciences, Southern Medical University, Guangzhou, 510515 Guangdong China

**Retraction Note: J Exp Clin Cancer Res 35, 152 (2016)**

**https://doi.org/10.1186/s13046-016-0426-8**

The Editor-in-Chief has retracted this article at the request of Wen-Ting Liao. The author stated that the data presented in Fig. 2F (HE and Ki67) were published in the authors’ previous study [1], and the data in Figs. 1C (α-tubulin, FOXO3, p-AKT) and 2A (α-tubulin) were published in [2]. In addition, the same α-tubulin western blot bands are presented in Figs. 2A and 3A. Thus, the corresponding author stated that the authors have no confidence in the reliability of the data and conclusions reported in this article. Run-Wei Yang, Ying-Yue Zeng, Wen-Ting Wei, Yan-Mei Cui, Hui-Ying Sun, Li Liang, and Wen-Ting Liao agree to this retraction. Yue-Long Cai, Xin-Xin Nian, Yun-Teng Hu, Yu-Ping Quan, Sheng-Lu Jiang, Meng Wang, Ya-Li Zhao, Jun-Feng Qiu, Ming-Xuan Li, Jia-Huan Zhang, Mei-Rong He, and Yan-Qing Ding have not responded to any correspondence about this retraction.
